# Detection of histone H3 K27M mutation and post-translational modifications in pediatric diffuse midline glioma via tissue immunohistochemistry informs diagnosis and clinical outcomes

**DOI:** 10.18632/oncotarget.26430

**Published:** 2018-12-14

**Authors:** Tina Huang, Roxanna Garcia, Jin Qi, Rishi Lulla, Craig Horbinski, Amir Behdad, Nitin Wadhwani, Ali Shilatifard, Charles James, Amanda Saratsis

**Affiliations:** ^1^ Division of Pediatric Neurosurgery, Department of Surgery, Ann & Robert H. Lurie Children's Hospital of Chicago, Chicago, IL, USA; ^2^ Department of Neurological Surgery, Northwestern University Feinberg School of Medicine, Chicago, IL, USA; ^3^ Division of Pediatric Hematology/Oncology, Hasbro Children's Hospital, The Warren Alpert Medical School of Brown University, Providence, RI, USA; ^4^ Department of Pathology, Northwestern University Feinberg School of Medicine, Chicago, IL, USA; ^5^ Department of Pathology, Ann & Robert H. Lurie Children's Hospital of Chicago, Chicago, IL, USA; ^6^ Department of Biochemistry and Molecular Genetics, Northwestern University Feinberg School of Medicine, Chicago, IL, USA

**Keywords:** diffuse midline glioma, pediatric glioma, diffuse intrinsic pontine glioma, histone H3K27M mutation, histone H3 post-translational modification

## Abstract

Pediatric diffuse midline glioma is a highly morbid glial neoplasm that may arise in the thalamus or brainstem (also known as diffuse intrinsic pontine glioma or DIPG). Because tumor anatomic location precludes surgical resection, diagnosis and treatment is based on MR imaging and analysis of biopsy specimens. Up to 80% of pediatric diffuse midline gliomas harbor a histone H3 mutation resulting in the replacement of lysine 27 with methionine (K27M) in genes encoding histone H3 variant H3.3 (*H3F3A*) or H3.1 (*HIST1H3B*). H3K27M mutant glioma responds more poorly to treatment and is associated with worse clinical outcome than wild-type tumors, so mutation detection is now diagnostic for a new clinical entity, diffuse midline glioma H3K27M mutant, as defined in the most recent WHO classification system. We previously reported patterns of histone H3 trimethylation (H3K27me3) and acetylation (H3K27Ac) associated with H3K27M mutation that impact transcription regulation and contribute to tumorigenesis. Given the clinical implications of the H3K27M mutation and these associated H3 post-translational modifications (PTMs), we set to determine whether they can be characterized via immunohistochemistry (IHC) in a cohort of pediatric glioma (*n* = 69) and normal brain tissue (*n* = 4) specimens. We observed 100% concordance between tissue IHC and molecular sequencing for detecting H3K27M mutation. In turn, H3K37M and H3K27me3 results, but not H3K27Ac staining patterns, were predictive of clinical outcomes. Our results demonstrate H3K27M and H3K27me3 staining of pediatric glioma tissue may be useful for diagnosis, stratification to epigenetic targeted therapies, and longitudinal monitoring of treatment response.

## INTRODUCTION

**Figure 1:**
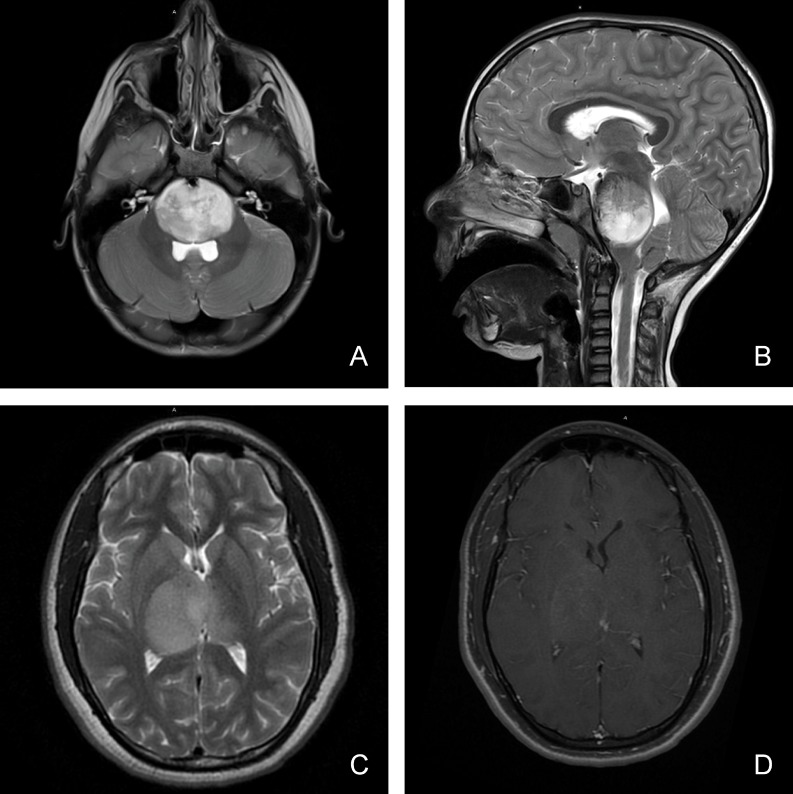
Magnetic resonance imaging characteristics (MRI) of pediatric diffuse midline glioma Pediatric diffuse midline glioma arise in the brainstem (**A**, **B**) or thalamus (**C**, **D**). T2 weighted MR imaging (A, B, C) demonstrates a homogenous, expansile, infiltrative lesion with extension within white matter tracts. Minimal to heterogenous enhancement may be observed on T1 post-gadolinium sequences (D). Local mass effect and perilesional edema may also be seen.

Pediatric high-grade glioma (HGG) persists as the leading cause of cancer death in children. A subset of pediatric HGG, known as diffuse midline glioma, presents a particularly significant diagnostic and therapeutic challenge due to its midline location, infiltrative nature, and aggressive tumor biology (Figure 1). Somatic mutations in histone 3 (H3) isoforms H3.3 and H3.1, resulting in substitution of methionine for lysine at residue 27 of the histone H3 N-terminal tail (p.Lys27Met or K27M), are detected in >80% of diffuse midline gliomas, including thalamic glioma and diffuse intrinsic pontine glioma (DIPG) [1–4]. Due to this change in the amino acid sequence of the N-terminal tail of Histone H3, H3K27M mutant tumors also harbor altered patterns of histone H3 post-translational modification (PTM). For example, a reduction in polycomb repressive complex 2 (PRC2) mediated genomic H3K27 trimethylation (H3K27me3) has been shown in H3K27M glioma [3, 4]. We also recently demonstrated genomic co-enrichment of acetylated H3K27 (H3K27ac) with H3K27M mutant protein in H3K27M mutant DIPG cells, with exclusion of PRC2 complex subunits SUZ12 and EZH2 [3, 4]. Since these H3K27M-associated changes in H3 marks significantly affect chromatin structure and function, resulting in distinct gene and protein expression, as well as DNA methylation patterns [1, 2, 5–8] and tumor phenotype [1, 3, 4, 9, 10], detection of changes in H3K27me3 and H3K27Ac over time may be important for assessing tumor response to epigenetic therapies.

Importantly, patients with H3K27M mutant glioma have poorer overall response to therapy and clinical outcomes relative to H3 wild-type. As a result of these significant implications on tumor biology and patient clinical outcomes, molecular detection of the H3K27M mutation in thalamic or brainstem glioma is now diagnostic for a new clinical entity known as diffuse midline glioma K27M-mutant, which receives a WHO grade IV designation regardless of tissue histological features [11]. While DNA sequencing remains the gold standard for H3K27M detection, a technique for identification via immunohistochemical (IHC) staining of formalin fixed paraffin-embedded (FFPE) pediatric glioma tissue was recently reported [12, 13]. This represents an important diagnostic achievement due to the relative efficiency and cost effectiveness of IHC staining compared to molecular sequencing analysis. In addition, diffuse midline glioma tissue is typically obtained via stereotactic tumor biopsy, limiting the amount specimen available for molecular analysis [14, 15]. While detection of H3K27M mutant and associated reduction of H3K27me3 is reported [16], H3K27Ac co-enrichment in H3K27M mutant tumors was not previously explored. To the best of our knowledge, we are the first to examine the clinical utility of tissue IHC to characterize H3K27Ac and H3K27me3 expression patterns in a large cohort of pediatric glioma tissue specimens (*n* = 69), and to further analyze these results with respect to tumor clinicopathologic features and clinical outcomes in order to determine the clinical utility of this approach.

## RESULTS

### Immunohistochemical staining

Demographic and descriptive statistical results for the cohort of cases analyzed are summarized in Table 1. This included 69 pediatric glioma tissue specimens from tumors arising in four neuroanatomic locations: brainstem (*n* = 18, 26%), thalamus (*n* = 27, 39%), cerebral hemisphere (*n* = 15, 21%) and cerebellar hemisphere (*n* = 9, 13%). Four normal brainstem tissue specimens were also analyzed as controls. Subject age ranged from nine months to 20 years (mean 49 months, standard error of the mean (SEM) = 7.2 months). Female and male genders were similarly represented in the cohort (51% and 49%, respectively). The majority of specimens (56%) were high-grade, with 20% designated Grade III and 36% Grade IV by histopathological features.

**Table 1:** Summary of pediatric glioma tissue specimens analyzed

	Tumor tissue (*n* = 69)	Normal tissue (*n* = 4)
**Gender**		
** Male**	34 (49.28)	2 (50.00)
** Female**	35 (50.72)	2 (50.00)
**Age**		
** <5 years**	14 (20.29)	3 (75.00)
** 5–10 years**	26 (37.68)	1 (25.00)
** >10 years**	29 (42.03)	0 (0.00)
**Anatomical location**		
**Midline**		
** Brainstem**	18 (26.09)	4 (100.00)
** Thalamus**	27 (39.13)	0 (0.00)
**Non-midline**		
** Cerebellar hemisphere**	9 (13.04)	0 (0.00)
** Cerebral hemisphere**	15 (21.74)	0 (0.00)
**WHO-Grade**		
** I**	20 (28.99)	n/a
** II**	10 (14.49)	n/a
** III**	14 (20.29)	n/a
** IV**	25 (36.23)	n/a

Formalin fixed paraffin embedded pediatric glioma and normal brainstem tissue specimens were submitted for immunohistochemical (IHC) analysis. Gender, age, tumor anatomical location and WHO-grade of specimens are summarized. KEY: Number in parentheses indicate percentage of total cohort.

**Figure 2:**
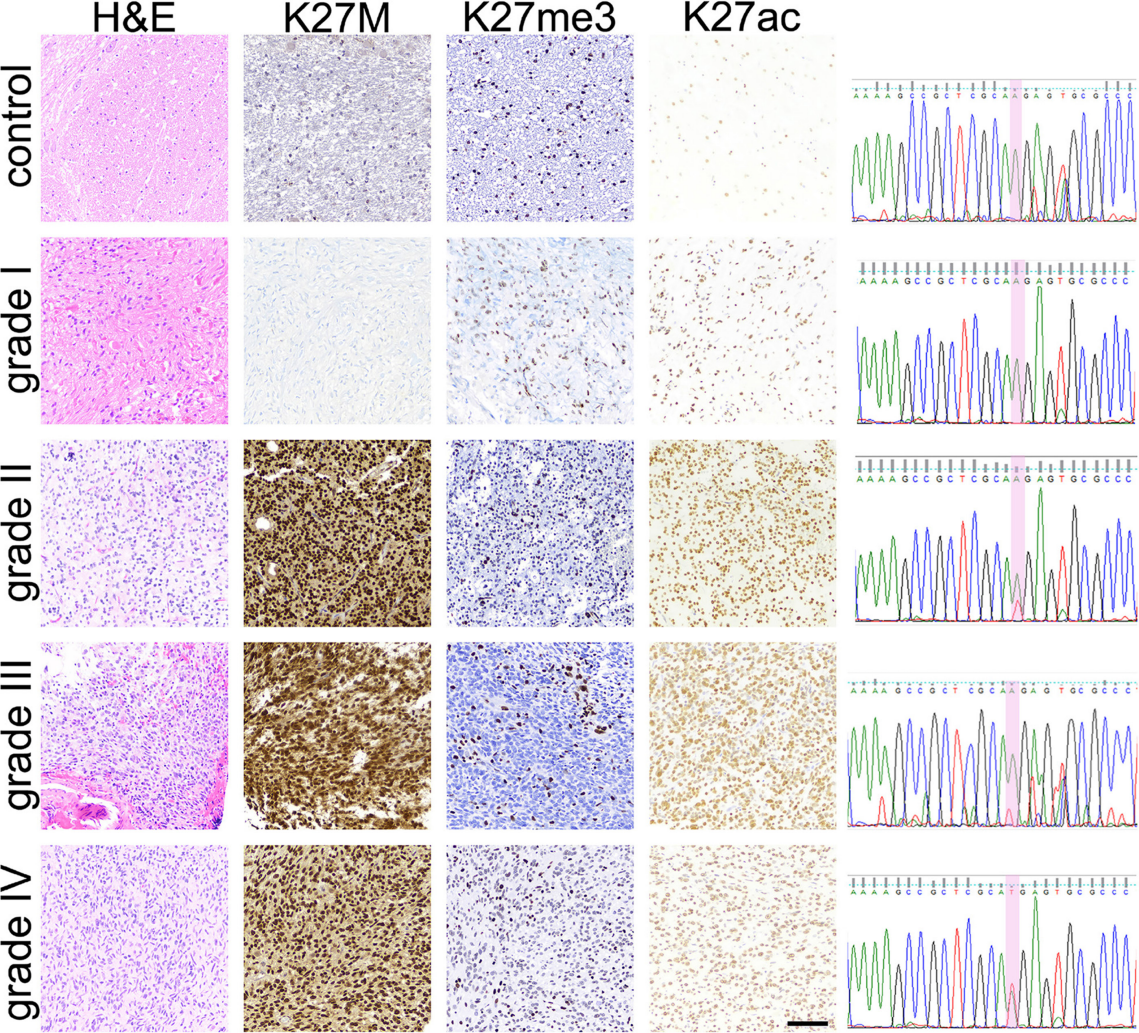
Pediatric glioma tissue immunohistochemical (IHC) staining results IHC staining results for H3K27M, H3K27me3 and H3K27Ac in WHO-grade I-IV tumor tissue and normal brainstem tissue specimens are depicted. Hematoxylin and Eosin staining was used to confirm tissue diagnosis of glioma and of WHO-grade. H3K27M IHC results were verified via PCR-amplification of a 300bp region of *H3F3A* followed by Sanger sequencing, with 100% concordance between molecular and IHC mutation detection. c.83A>T transversion (red peak) was detected in grade II, III, and IV tumors. No mutation was detected in brainstem control or grade I tumor tissue. Scale bar = 100 microns.

All tumor tissue (*n* = 69) and normal brain tissue (*n* = 4) was stained for H3K27M. In addition, 61 glioma cases in our cohort were stained for H3K27me3, and 33 for H3K27Ac. Immunohistochemical staining results for H3K27M and H3K27me3 across our cohort were consistent with prior investigations and reports in the literature (Figure 2). H3K27M staining revealed 38 mutant (55%) and 31 wild-type (45%) tissue specimens (Table 2). All normal brainstem specimens were H3K27 wild-type. The presence of H3K27M mutation was significant by tumor location (*p* < 0.001), with midline tumors more likely to harbor the mutation compared to those arising in the cerebral or cerebellar hemisphere (*p* = 0.012). Fifty percent of our H3K27M mutant cases occurred in children between 5 and 10 years of age. There was no relationship between H3K27M and tumor histopathological grade (*p* = 0.594) or gender (*p* = 0.23). H3K27me3 signals significantly differed by tumor grade (*p* < 0.02), with greater H3K27me3 loss in high-grade tumors (Grade III and IV) tumors relative to low-grade lesions (I and II, *p* = 0.016). Midline tumors also demonstrated significantly lower H3K27me3 signals compared to non-midline tumors (*p* = 0.036). No relationship was observed between H3K27me3 signal intensity and gender (*p* = 0.59) or age (*p* = 0.62). As expected, H3K27M mutation correlated with reduced H3K27me3 signal (R = −0.32, *p* = 0.01). In contrast, there was no association of H3K27Ac positivity with H3K27M status (R = 0.17, *p* = 0.32). There was no relationship between H3K27me3 and H3K27Ac detection either by direct comparison (*p* = 0.86) or when parsed by degree of H3K27Ac signal intensity (*p* = 1.00). Similarly, there was no relationship between H3K27me3 and H3K27Ac positivity when evaluated by tumor anatomic location (brainstem R = 0.31, *p* = 0.15; non-brainstem R = −0.53, *p* = 0.14).

**Table 2:** Descriptive statistical analysis of H3K27M, H3K27me3, and H3K27Ac immunohistochemical (IHC) staining results

	H3K27M (*n* = 69)			H3K27me3 (*n* = 61)			H3K27Ac (*n* = 33)			
	MT = 38	WT = 31	*p*-value	Present = 40	Absent = 21	*p*-value	High = 24	Medium = 4	Low = 5	*p*-value
**Age**			0.008^**^			0.62				0.626
** <5 years**	9 (23.68)	8 (25.81)		13 (32.50)	5 (23.81)		11 (45.83)	1 (25.00)	1 (20.00)	
** 5–10 years**	19 (50.00)	4 (12.90)		10 (25.00)	7 (33.33)		5 (20.83)	1 (25.00)	3 (60.00)	
** >10 years**	10 (26.32)	19 (61.29)		17 (42.50)	9 (42.86)		8 (33.33)	2 (50.00)	1 (20.00)	
**WHO-Grade**			0.594			0.02^*^				0.782
** I**	11 (28.95)	9 (29.03)		18 (45.00)	2 (9.52)		7 (29.17)	1 (25.00)	1 (20.00)	
** II**	4 (10.53)	6 (19.35)		5 (12.50)	3 (14.29)		2 (8.33)	1 (25.00)	0 (0.00)	
** III**	7 (18.42)	7 (22.58)		8 (20.00)	5 (23.81)	High Grade 0.016^*^	4 (16.67)	1 (25.00)	2 (40.00)	
** IV**	16 (42.11)	9 (29.03)		9 (22.50)	11 (52.38)		11 (45.83)	1 (25.00)	2 (40.00)	
**Gender**			0.230			0.59				0.025^*^
** Male**	16 (42.11)	18 (58.06)		17 (42.50)	11 (52.38)		8 (33.33)	4 (100.00)	3 (60.00)	
** Female**	22 (57.89)	13 (41.94)		23 (57.50)	10 (47.62)		16 (66.67)	0 (0.00)	2 (40.00)	
**Anatomical Location**			0.001^***^			1				1
**Midline**			Midline 0.012^*^			Midline 0.036^*^				
** Brainstem**	16 (42.11)	2 (6.45)		8 (20.00)	9 (42.86)		7 (29.17)	1 (25.00)	1 (20.00)	
** Thalamus**	15 (39.47)	12 (38.71)		17 (42.50)	10 (47.62)		16 (66.67)	3 (75.00)	4 (80.00)	
**Non-midline**										
**Cerebellar Hemisphere**	3 (7.89)	6 (19.35)		6 (15.00)	1 (4.76)		0 (0.00)	0 (0.00)	0 (0.00)	
**Cerebral Hemisphere**	4 (10.53)	11 (35.48)		9 (22.50)	1 (4.76)		1 (4.17)	0 (0.00)	0 (0.00)	
**Progression**	(*n* = 65)		0.397	(*n* = 57)		0.51		(*n* = 32)		0.643
** Yes**	28 (80.00)	21 (70.00)		28 (75.68)	17 (85.00)		21 (91.30)	4 (100.00)	4 (80.00)	
** No**	7 (20.00)	9 (30.00)		9 (24.32)	3 (15.00)		2 (8.07)	0 (0.00)	1 (20.00)	
**Death**	(*n* = 63)		0.044^*^	(*n* = 55)		<0.001^***^		(*n* = 30)		1
** Yes**	20 (60.61)	10 (33.33)		12 (32.43)	16 (88.89)		14 (67.67)	3 (75.00)	3 (60.00)	
** No**	13 (39.39)	20 (60.67)		25 (67.57)	2 (11.11)		7 (33.33)	1 (25.00)	2 (40.00)	
**Recurrence**	(*n* = 17)		0.637	(*n* = 14)		1		(*n* = 3)		n/a
** Yes**	3 (17.65)	6 (35.29)		6 (54.55)	1 (33.33)		2 (67.33)	No data	No data	
** No**	4 (23.53)	4 (23.53)		5 (45.45)	2 (66.67)		1 (33.33)			

Patient clinical characteristics and outcomes relative to tumor Histone H3K27M, H3K27me3, H2K27Ac staining results. Between groups comparison was performed using Fischer's exact test for statistical significance. KEY: MT = H3K27M positive, WT = H3K27M negative. ^*^= statistical significance: ^*^p ≤ 0.05, ^**^p ≤ 0.01, ^**^p ≤ 0.001, Fischer's exact test. Average H3K27Ac staining score ranked low (0-33%), medium (33.1-66%), and high (66.1-100%). Numbers in parentheses indicate percentage of total cohort.

### Mutation validation via sanger sequencing

To validate tissue immunohistochemical detection of H3K27M mutation, we analyzed one fresh frozen tumor tissue representing each WHO-grade (WHO-grade I–IV, *n* = 4), and one normal brainstem specimen as a negative control, via PCR-amplification and Sanger sequencing of the *H3F3A* gene (Figure 2). An c.83A>T transversion resulting in H3K27M mutation was detected in Grade II, III and IV specimens, but not in the normal brainstem or Grade I tumor tissue, yielding 100% concordance between molecular and IHC data.

### Clinical outcomes

**Figure 3:**
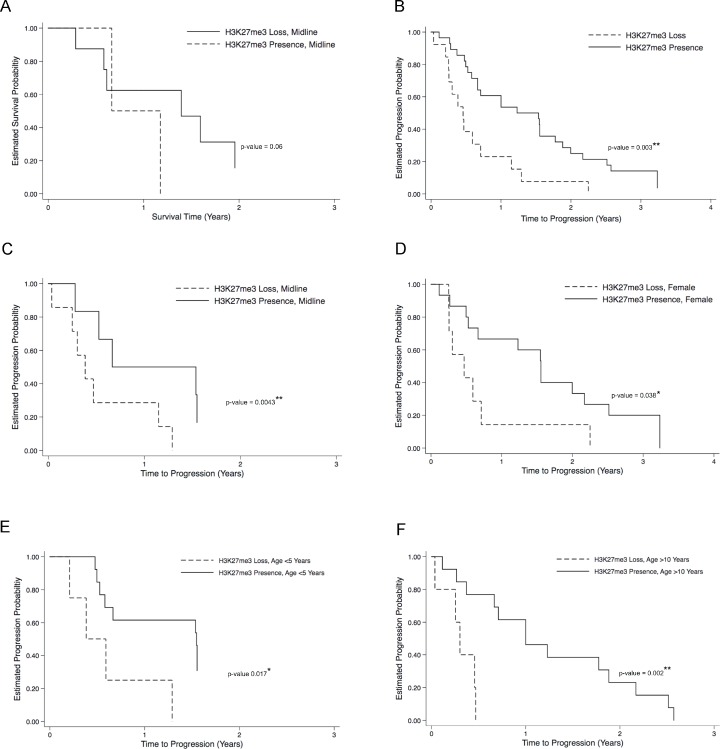
H3K27me3 loss correlates to poor overall clinical outcome in H3K27M pediatric glioma Kaplan–Meier plots for progression-free survival (PFS) and disease progression curves are illustrated between patients with and without H3K37me3 loss. Even though there were no overall significant likelihood of death associated with H3K27me3 loss, neared significant likelihood of death was observed when tumors are midline (**A**). There was significant likelihood of disease progression associated with H3K37me3 loss (**B**), especially when tumors are midline (**C**), in female patients (**D**), and when patients are less than five years old (**E**), or more than ten years old (**F**). ^*^p < 0.05, ^**^p < 0.01, log rank test.

Long-term survival data was available for 63 glioma patients in our cohort (Tables 2 and 3A). These data were examined for a relationship with H3K27M, H3K27me3 and H3K27Ac staining results. Twenty-one of the 33 patients in our cohort with H3K27M positive glioma have died. Overall, there was a greater incidence of death in patients with H3K27M mutation relative to wild-type (*p* < 0.044, Table 2). There was no difference in mortality risk amongst H3K27M mutant tumors when parsed by tumor location, gender, or designated WHO grade (Table 3A). However, a significant risk of increased mortality amongst H3K27M mutant tumors was observed in patients less than five years old (*p* = 0.035), with a trend towards increased risk of mortality in patients 5–10 years of age (*p* = 0.06) tumors. Overall, we also observed increased risk of mortality with H3K27me3 loss (*p* < 0.001, Table 2). Amongst cases with loss of H3K27me3, risk of death was significantly increased when detected in midline tumors (*p* = 0.06, Figure 3A), with no significance when analyzed by patient age, gender or WHO-grade on multivariable analysis. In contrast, H3K27Ac staining results did not inform on likelihood of death on direct comparison or log-rank testing.

**Table 3A:** Log rank test for survival

	H3K27 Mutant			H3K27me3-			H3K27Ac				
								Low	Medium	High	
	*n*	# of deaths	*p*-value	*n*	# of deaths	*p*-value	*n*	# of deaths			*p*-value
**Total**	33	21	0.283	27	15	0.798	30	3	3	14	0.294
**Anatomical location**											
**Midline**	23	17	0.551	22	15	0.060	19	3	3	13	0.197
** Brainstem**	9	9	n/a	8	6	0.371	6	1	1	4	0.556
** Thalamus**	14	8	0.631	14	9	0.762	13	2	2	9	0.212
**Non-midline**	6	2	0.198	5	0	n/a	1	n/a	n/a	n/a	n/a
** Cerebellar hemisphere**	1	1	n/a	1	0	n/a	n/a	n/a	n/a	n/a	n/a
** Cerebral hemisphere**	5	1	0.156	4	0	n/a	1	n/a	n/a	1	n/a
**Gender**											
** Female**	20	11	0.521	13	7	0.636	10	1	n/a	9	0.963
** Male**	13	9	0.346	14	8	0.729	10	1	3	5	0.187
**Age**											
** <5 years**	9	5	0.035^*^	7	4	0.446	7	n/a	1	6	0.177
** 5-10 years**	6	6	0.060	6	4	0.761	5	2	1	2	0.864
** >10 years**	14	8	0.598	14	7	0.295	8	1	1	6	0.253
**WHO-Grade**											
** I**	10	0	n/a	1	0	n/a	3	n/a	n/a	1	n/a
** II**	3	2	0.157	3	2	0.225	2	n/a	1	1	0.317
** III**	10	6	0.656	10	5	0.523	6	1	1	4	0.276
** IV**	15	11	0.321	13	8	0.972	11	2	1	8	0.857

*n* = 63, total deaths = 33 (52.38%).

Association between IHC staining patterns and clinical disease progression was also evaluated in cases for which this data was available (*n* = 65, Tables 2 and 3A). Of this subset, 49 patients (75.38%) demonstrated disease progression (mean 23.34 months, SEM = 16.24, range 1.6 to 20 years). There was no difference in incidence of disease progression between H3K27M mutant and wild-type cases (*p* < 0.397, Table 2). Amongst patients with H3K27M mutation, there was no difference in likelihood of disease progression across tumor location, gender, age or WHO grade. In contrast, amongst cases with H3K27me3 signal loss, a significant likelihood of disease progression was observed on log-rank testing (*p* = 0.003, Figure 3B), amongst midline tumors (*p* < 0.0043, Figure 3C), in females (*p* = 0.038, Figure 3D), children less than 5 years of age (*p* = 0.017, Figure 3E), children more than 10 years of age (*p* = 0.002, Figure 3F), and near significance in children with WHO-grade IV tumors (*p* = 0.065, Table 3B). Again, H3K27Ac staining intensity did not inform on risk of disease progression, though we did observe increased likelihood of disease progression with high H3K27Ac in the subset of children under 5 years of age (*p* = 0.002, Table 3B). To confirm that the H3K27Ac IHC staining is accurately reflecting overall acetylation status, we performed western blot analysis on tumor tissue derived total protein using the same antibody employed for IHC (Supplementary Figure 1A). Densitometry analysis of resulting bands demonstrated significantly greater relative H3K27Ac staining in tumor samples 1–3, all which were assigned a high H3K27Ac IHC score (3), compared to sample 4 with a low H3K27Ac IHC score (1, Supplementary Figure 1B, *p* = 0.0357).

**Table 3B:** Log rank test for progression

	H3K27 Mutant			H3K27me3-			H3K27Ac				
								Low	Medium	High	
	*n*	# of events	*p*-value	*n*	# of events	*p*-value	*n*	# of events			*p*-value
**Total**	45	25	0.870	41	13	0.003^**^	25	2	4	19	0.171
**Anatomical location**											
**Midline**	33	23	0.784	32	13	0.0043^**^	*25*	2	4	19	0.171
** Brainstem**	14	12	0.201	13	7	0.056	7	1	1	5	0.401
** Thalamus**	19	11	0.664	19	6	0.062	18	1	3	14	0.220
**Non-midline**	12	2	0.887	9	0	n/a	n/a	n/a	n/a	n/a	n/a
** Cerebellar hemisphere**	5	1	0.107	4	0	n/a	n/a	n/a	n/a	n/a	n/a
** Cerebral hemisphere**	7	1	0.758	5	0	n/a	n/a	n/a	n/a	n/a	n/a
**Gender**											
** Female**	23	14	0.598	22	7	0.038^*^	14	n/a	n/a	14	n/a
** Male**	22	11	0.706	19	6	0.065	11	2	4	5	0.225
**Age**											
** <5 years**	18	11	0.557	17	4	0.017^*^	12	1	1	10	0.002^**^
** 5-10 years**	6	6	n/a	6	4	0.863	5	1	1	3	0.641
** >10 years**	21	8	0.188	18	5	0.002^**^	8	n/a	2	6	0.775
**WHO-Grade**											
** I**	10	4	0.411	10	0	n/a	7	n/a	1	6	0.701
** II**	8	3	0.520	6	2	0.863	3	n/a	1	2	0.157
** III**	11	7	0.151	11	4	0.481	6	1	1	4	0.381
** IV**	16	11	0.823	14	7	0.065	9	1	1	7	0.543

*n* = 65, total progression = 49 (75.78%).

^*^= statistical significance: ^*^p ≤ 0.05, ^**^p ≤ 0.01, Log rank test.

## DISCUSSION

Somatic missense mutations in Histone H3.3 and H3.1 genes (*H3F3A* and *HIST1H3B*) occur in up to 80% of pediatric diffuse midline gliomas, including thalamic and diffuse intrinsic pontine gliomas (DIPGs). This mutation causes Lys27Met (K27M) substitution at a critical regulatory location on the Histone H3 N-terminal tail, affecting chromatin structure and regulation of gene transcription [1, 10, 17]. H3K27M mutant gliomas are known to exhibit distinct gene and protein expression, and have poorer overall survival and response to treatment relative to H3K27 wild-type tumors, independent of traditional tissue histopathologic features [1, 2, 5, 6, 18]. Because H3K27M mutation detection is more predictive of patient clinical outcome than traditional tumor morphologic features, molecular diagnosis of mutation status is now required for all cases of diffuse midline glioma, and K27M mutant tumors are deemed WHO Grade IV irrespective of tumor histopathologic features [11]. In addition, the H2K27M is associated with specific changes in H3 post-translational modifications (PTMs), including H3K27me3 loss and H3K27Ac gain in distinct genomic regions. However, these tumors are usually not amenable to surgical removal due to their deep anatomic location (Figure 1), so often only advanced biopsy is performed, limiting the amount of tissue available for mutation and associated H3 PTM characterization via advanced molecular techniques. Multiple reports have demonstrated the clinical feasibility of H3K27M mutation detection via tissue IHC [12–15]. To our best knowledge, there are no prior studies examining the clinical utility of tissue IHC for characterizing Histone H3 PTM states associated with H3K27M mutation in pediatric diffuse midline glioma.

Our group recently identified H3K27M-K27ac heterotypic nucleosomes in H3K27M mutant DIPG cell lines, suggesting that the H3K27M mutant protein may prevent H3K27 trimethylation (H3K27me3) via exclusion of PRC2 subunits EZH2 and SUZ12 from chromatin binding. This exclusion permits H3K27 acetylation (H3K27Ac), promoting active transcription at specific loci throughout the genome that contributes to tumorigenesis and is the basis for investigation of novel epigenetic targeted therapies for H3K27M mutant tumors [4]. We also recently demonstrated preclinical efficacy of BET/Bromodomain inhibition for DIPG treatment, while Mohammad and colleagues showed that inhibition of EZH2, the enzymatic subunit of PRC2 that catalyzes H3K27 trimethylation (H3K27me3), may also be an effective therapeutic strategy for treating H3K27M DIPG [3]. Since both BET and EZH2 inhibition result in increased H3K27me3 and decreased H3K27Ac, detection of these changes in H3 PTM over time may be useful for assessing tumor response to epigenetic therapies. For example, in our mouse xenograft of human DIPG we have shown decreased H3K27me3 and increased H3K27Ac staining of tumor tissue is associated with clinical response to BET / Bromodomain inhibition [4]. Additional epigenetic therapeutic strategies targeting the H3K27M mutation and its effects are currently under investigation in multiple clinical trials, with promising early results [19, 20], and response to these therapies may also be associated with changes in the Histone H3 modification landscape. Therefore, we set to characterize H3K27M mutation status and associated changes in H3K27me3 and H3K27Ac via tissue IHC in a large cohort of pediatric glioma tissue in order to determine the clinical utility of this approach for tumor epigenetic analysis.

In our cohort of 69 pediatric gliomas, 55% were H3K27M positive by IHC staining (Table 2). Importantly, we validated our IHC results via DNA Sanger sequencing of a subset of these tumors, with 100% concordance between molecular and IHC diagnosis (Figure 2). As expected, midline tumors were more likely to harbor the H3K27M mutation, comparable to prior studies [21]. H3K27M mutation was associated with greater risk of death amongst children <5 years old, and neared significance for children between five and 10 years old, the typical age for diffuse midline glioma diagnosis.

Importantly, H3K27M mutation also was associated with a higher likelihood of death, independent of tumor location and histologic grade, similar to findings recently reported by Pratt and colleagues [15, 22]. The findings reported here provide further evidence that the H3K27M mutation in pediatric glioma, as detected via tissue IHC, is a better prognostic indicator than traditional tumor histopathologic grading. Among our cohort of H3K27M mutant cases, poor clinical outcome in children with midline tumors was not related to tumor histological grade (Table 3). While we expect tumors designated as WHO-IV by conventional morphologic grading characteristics would demonstrate poor clinical outcome, independent of H3K27M mutation status, multiple groups have reported significant intratumoral heterogeneity in diffuse midline glioma that could explain this observation in our cohort. For example, Bugiani and colleagues recently performed postmortem analysis of nine DIPGs with known H3K27M mutation status and noted marked intratumoral heterogeneity [23]. In their series, five H3K27M mutant tumors demonstrated focal regions of WHO grade I morphology, and two H3K27 wild-type tumors demonstrated H3K27me3 immunonegativity. Taken together, these results suggest the potential risk of under-scoring tumor morphologic grade, which may skew analytic results. Further, that H3K27M mutation status supersedes tumor morphologic features in predicting clinical outcomes is the premise underlying the new WHO diagnostic criteria for diffuse midline glioma K27M mutant, and indeed the clinical implications of this molecular diagnosis is supported in the present study.

Importantly, we detected decreased H3K27me3 staining in H3K27M mutant tumors compared to wild-type (56% vs. 85%, respectively). This finding is in line with our published cell line data and other studies that demonstrate loss of H3K27 trimethylation in the setting of H3K27M mutation [3, 4, 9, 10, 12, 16, 17, 22, 24]. In turn, H3K27me3 loss was associated with poorer overall survival as well as greater likelihood of disease progression in our patient cohort (Table 3), most significantly among midline tumors and patient younger than 5 or over 10 years old, again consistent with prior reports [1, 2, 10, 20, 22, 25]. This is an important finding given the relative clinical ease of H3K27me3 detection via IHC staining, with the potential for utilization as a marker of response to epigenetic and other novel therapeutic strategies. In contrast, H3K27Ac staining results were less clinically informative. While H3K27Ac expression was detectable by IHC, these results were more difficult to quantify than H3K27me3 staining patterns, and therefore may have less clinical utility. Using two different scoring methods across three independent pathologists, we found no association between H3K27Ac staining intensity and either H3K27me3 or H3K27M status in our cohort. H3K27Ac signal intensity did not inversely correlate with H3K27me3 staining patterns as expected based on our prior cell and animal studies [4]. H3K27Ac positivity did correlate with disease progression among children less than five years old, suggesting clinical detection of changes in H3K27Ac staining patterns in response to targeted therapy may be of utility in only a small subset of diffuse midline glioma patients. Further studies are currently underway to optimize H3K27Ac staining protocols in additional mouse and human glioma tissues and validate these findings in a larger cohort.

We demonstrate in this study the clinical feasibility and utility of IHC staining for H3K27M mutation and two associated H3 post-translational modifications, H3K27me3 and H3K27Ac, in pediatric glioma. Recent advances in neurosurgical techniques now enable safe stereotactic biopsy of brainstem and thalamic gliomas [26]. As a result, diagnostic tumor biopsy of diffuse midline gliomas is increasingly performed for molecular analysis (Histone H3.3 and H3.1 sequencing) and treatment stratification. Furthermore, some H3 mutant tumors may be at least partially resectable, making serial tissue monitoring possible in select cases of tumor progression or recurrence. Given the high morbidity of these tumors and relative safety of stereotactic tumor biopsy, serial tissue sampling for measuring response to epigenetic-targeted therapies for diffuse midline glioma is no longer outside the realm of clinical feasibility. As previous reports have demonstrated H3K27me3 loss in association with the H3K27M mutation [16], our findings validate the accuracy of tissue IHC for characterizing H3 post-translational modifications in H3K27M mutant tumors and informing clinical outcomes. These results suggest the potential utility of IHC staining for longitudinal monitoring of response to novel, epigenetic targeted therapies.

In our cohort, H3K27M mutation was the strongest predictor of clinical outcome in our cohort, independent of tumor grade or location, while H3K27me3 negativity, but not H3K27ac positivity, was predictive of clinical outcomes. While H3K27M status will not change in response to epigenetic therapies, the proportion of H3K27me3 and H3K27Ac may. Our results suggest that monitoring tumor H3K27me3 levels vial simple IHC could be useful for stratification to epigenetic therapies, and evaluating therapeutic response. As H3K27me3 loss was not consistently accompanied by a reciprocal gain in H3K27Ac staining, our results suggest H3K27Ac detection via IHC may not be as useful as predicted by our preclinical studies.

Overall, given the relative lack of diffuse midline glioma tumor tissue for advanced molecular analysis compared to other brain tumors that are anatomically accessible for surgical removal, our study suggests that tissue IHC staining for H3K27M and H3K27me3 may serve as a cost-effective and efficient way to evaluate the efficacy of promising new therapies on the tumor epigenetic landscape, with the potential to portend clinical outcomes for these patients. In addition, we and others have shown detection of tumor proteins in CSF [27, 28], and are working to characterize histone proteoforms including H3K27 methylation and acetylation states, with the goal of determining if changes to these histone marks can be detected over time. The ability to correlate longitudinal changes in these marks in CSF, a clinically more accessible substrate that midline glioma tissue, with H3K27 status as informed by IHC from small tissue specimens, could pave the way for developing less invasive means of molecular analysis, making IHC staining a valuable test at this time. To further explore these relationships, whole transcriptome and ChIP-Seq analysis of pediatric glioma tissue in a subset of these H3K27M tumors is currently underway. In addition, our group is currently evaluating these epigenetic marks via tissue IHC in response to novel epigenetic therapies in preclinical models of DIPG, and this approach should be considered when investigating new agents for therapeutic trial.

## MATERIALS AND METHODS

### Tissue specimens and institutional approvals

Tissue specimens from children with glioma treated at our institution between 2004–2014 were collected during the course of treatment (*n* = 59) or post mortem (*n* = 14), and preserved via formalin fixation and paraffin embedding (Table 1). This included 69 glioma tissue specimens, and four normal brainstem tissue specimens as controls. Five matched fresh frozen tissue specimens also collected during the course of treatment or post mortem (tumor *n* = 4, normal *n* = 1) were also analyzed. Informed consent for specimen collection and analysis was obtained under protocols approved by Ann & Robert H. Lurie Children's Hospital of Chicago and Northwestern University Institutional Review Boards (Lurie 2012-14877 and 2005-12252, NU STU00202063). All patient identifiers were removed at the time of specimen analysis, and a numerical identifier was assigned to each specimen before processing.

### Tissue immunohistochemistry

Tissue immunohistochemistry was performed as follows: serial sections of four microns in thickness were cut from paraffin blocks, deparaffinized with Xylene for 20 minutes, and rehydrated for three minutes in 100% ethanol, three minutes in 95% ethanol, and three minutes in 90% ethanol. For antigen retrieval, sections were washed in distilled water twice, transferred to 1× Dako Target Retrieval Solution (Dako S1699), and incubated in the Biocare Medical Decloaking Chamber for five minutes at 110° C, followed by incubation in PBS for five minutes. 200 μL peroxidase I (Dako K4011) were then added to the sections, incubate for ten minutes at room temperature, and rinse sections with PBS. Subsequently, two to five drops of background sniper (Biocare Medical BS966H) were added to the sections, incubate for fifteen minutes at room temperature, and rinse sections with PBS.

Primary antibodies were prepared to a final volume of 200 μL using antibody diluent (Dako S0809) as follows: rabbit polyclonal anti-Histone H3K27M (Millipore ABE419) 1:1000, rabbit monoclonal anti-Histone H3K27me3 (Cell Signaling Technology 9733) 1:200, and rabbit monoclonal and anti-Histone H3K27Ac (Cell Signaling Technology 8173S) 1:200. Tissue sections were incubated with primary antibody at 4° C overnight followed by wash in TBST/Tween (Dako S3306) for three minutes. Sections were then incubated with polymer-HRP anti-rabbit secondary antibody (Dako K4011) for 30 minutes, followed by PBS wash. Immunohistochemical reactions were visualized by incubating sections for three minutes in DAB chromogen (dilute one drop DAB into 1mL DAB buffer from Dako Kit K4011). The sections were then counter stained with hematoxylin for one minute at room temperature, washed with tap water and dehydrated with graded alcohol and xylene, and finally coverslip using a xylene-based mounting medium. Hematoxylin and Eosin (H&E) staining was performed on each specimen by the Pathology Department at the Ann & Robert H. Lurie Children's Hospital of Chicago in order to validate tissue diagnosis and assign WHO-grade.

Results of tissue IHC staining was evaluated independently by three pathologists, including one board certified neuropathologist, who were blinded to all specimen identifiers including histological diagnosis and anatomic tumor location at the time of tissue evaluation. H3K27M mutation and H3K27me3 positivity was defined as nuclear staining in >80% of tumor cells visualized in the absence of staining in tumor vascular epithelial cells (internal negative control), as previously described by Venneti *et al.* [22]. In cases where two individual pathological evaluations were not concordant, the third pathologist evaluation determined the final designation. The degree of H3K27Ac positivity was ranked on a scale of 0 (none observed) to 10 (present in all tumor cells), with the absence of non-specific vascular epithelial staining as an internal negative control. The average H3K27Ac staining score across ranking pathologists was then determined and converted to a percentage value, then stratified into groups as follows: Low 0–33%; Moderate 33.1–66%; High 66.1–100%.

### Clinicopathological data collection

A retrospective chart review of pediatric glioma patients from whom tissue specimens were collected and analyzed was performed to determine: patient gender; age at diagnosis; tumor anatomic location; tumor histologic diagnosis; tumor WHO-grade; date of surgery; extent of tumor resection; date of recurrent and/or progressive disease; date and nature of adjuvant therapy; overall survival; progression free survival; and mortality. Tumor recurrence was defined as MR- confirmed radiographic evidence of new disease burden after confirmation of gross total resection (GTR) of their tumor on initial post-operative MR imaging. Disease progression was defined as radiographic evidence of increased disease burden after tumor biopsy or subtotal resection (STR), or evidence of recurrent and increasing disease burden after GTR.

### Tissue mutation sequencing

Fresh frozen tumor tissue was used for DNA extraction using the DNeasy Blood & Tissue Kit (Qiagen) per manufacturer's protocol. Template DNA isolated from tumor tissue was amplified via PCR using H3F3A primers (0.8 mM) flanking a 300 base pair exonal region encoding Lys27 and Gly34 in Histone H3.3. Conventional PCR was performed in a thermocycler (Bio-Rad) under the following conditions: two minutes at 95° C, 40 cycles of (25 s at 95° C, 35 s at 55° C, 40 s at 72° C), and five minutes at 72° C. PCR products separated in 2% agarose gel and full-length H3F3A DNA purified using the QIAquick Gel Extraction Kit (Qiagen). Extracted DNA was submitted to Sanger sequencing of H3F3A for K27M mutation using the ABI 3730 High-Throughput DNA Sequencer (Applied Biosystems). Sequenced data were visualized with FinchTV (Geospiza) and MegAlign (DNASTAR).

### Statistical analysis

Study measures were estimated using *t*-tests and chi-square tests for continuous or dichotomous variables, respectively, to test the null hypothesis that patient outcomes, demographics or mutation status were the same across subgroups. When appropriate, the Fischer's exact test was implemented for categorical variables requiring small sample adjustment. Differences in survival and recurrence were analyzed using Kaplan–Meier method and the log-rank test was used to test the null hypothesis that the subgroups have identical recurrence or survival patterns. Patient data on follow-up time (in months) were compared for patients based on H3K27M mutation status (wild-type versus mutant), presence or absence of K27me3, or H3K27 acetylation expression (low, medium, or high) based on immunohistochemistry for the survival analysis. Statistical tests were considered significant for *p*-values < 0.05. Data were analyzed using Intercooled Stata, Version 14.0 (Stata Corp, College Station, TX).

### Western blotting

Frozen tissue was thawed and dissociated with gentleMACS Dissociator (130-093-235, Macs Miltenyi Biotec). Protein was extracted in Tissue Extraction Reagent (FNN071, Thermo Fisher Scientific) and concentration determined with Pierce BCA Protein Assay Kit (23225, Thermo Fisher Scientific). 60 μg of protein was separated by electrophoresis in a 4–15% precast protein gel (4561086, BioRad) and transferred to PVDF membranes. Blocking was subsequently performed with 5% non-fat milk in TBST, followed by incubation with anti-H3K27Ac antibody at 1:500 dilution (8173S, Cell Signaling Technology) overnight. After 5 washes with TBST, membranes were incubated with HRP-conjugated anti-Rabbit IgG antibody at 1:1000 (7074 Cell Signaling Technology) for 1 hour. Pierce ECL Plus (32132, Thermo Fisher Scientific) was used to detect protein bands. Blots were then stripped (46430, Thermo Fisher Scientific) and re-probed with anti-total H3 primary antibody at 1:1000 dilution (14269S, Cell Signaling Technology) as a loading control. HRP-conjugated anti-Mouse IgG antibody (7076, Cell Signaling Technology) was used to detect total H3 signal. Protein from a patient-derived DIPG cell line SF8628 was extracted with RIPA buffer (89900, Thermo Fisher Scientific) and processed in parallel with tissue specimens as a positive control. Densitometry analysis was performed with image J.

## SUPPLEMENTARY MATERIALS FIGURE



## Abbreviations

WHO: World Health Organization
(IHC): Immunohistochemistry
H3K27me3: H3K27 trimethylation
H3K27Ac: HeK27 acetylation
DIPG: Diffuse Intrinsic Pontine Glioma
GTR: Gross-total resection
STR: Sub-total resection
PTM: Post-translational Modification
PFS: Progression-Free Survival
SEM: Standard Error of the Mean.

## References

[1] Khuong-Quang DA, Buczkowicz P, Rakopoulos P, Liu XY, Fontebasso AM, Bouffet E, Bartels U, Albrecht S, Schwartzentruber J, Letourneau L, Bourgey M, Bourque G, Montpetit A (2012). K27M mutation in histone H3.3 defines clinically and biologically distinct subgroups of pediatric diffuse intrinsic pontine gliomas. Acta Neuropathol.

[2] Lulla RR, Saratsis AM, Hashizume R (2016). Mutations in chromatin machinery and pediatric high-grade glioma. Sci Adv.

[3] Mohammad F, Weissmann S, Leblanc B, Pandey DP, Hojfeldt JW, Comet I, Zheng C, Johansen JV, Rapin N, Porse BT, Tvardovskiy A, Jensen ON, Olaciregui NG (2017). EZH2 is a potential therapeutic target for H3K27M-mutant pediatric gliomas. Nat Med.

[4] Piunti A, Hashizume R, Morgan MA, Bartom ET, Horbinski CM, Marshall SA, Rendleman EJ, Ma Q, Takahashi YH, Woodfin AR, Misharin AV, Abshiru NA, Lulla RR (2017). Therapeutic targeting of polycomb and BET bromodomain proteins in diffuse intrinsic pontine gliomas. Nat Med.

[5] Saratsis AM, Kambhampati M, Snyder K, Yadavilli S, Devaney JM, Harmon B, Hall J, Raabe EH, An P, Weingart M, Rood BR, Magge SN, MacDonald TJ (2014). Comparative multidimensional molecular analyses of pediatric diffuse intrinsic pontine glioma reveals distinct molecular subtypes. Acta Neuropathol.

[6] Huang TY, Piunti A, Lulla RR, Qi J, Horbinski CM, Tomita T, James CD, Shilatifard A, Saratsis AM (2017). Detection of Histone H3 mutations in cerebrospinal fluid-derived tumor DNA from children with diffuse midline glioma. Acta Neuropathol Commun.

[7] Schwartzentruber J, Korshunov A, Liu XY, Jones DT, Pfaff E, Jacob K, Sturm D, Fontebasso AM, Quang DA, Tonjes M, Hovestadt V, Albrecht S, Kool M (2012). Driver mutations in histone H3.3 and chromatin remodelling genes in paediatric glioblastoma. Nature.

[8] Gielen GH, Gessi M, Hammes J, Kramm CM, Waha A, Pietsch T (2013). H3F3A K27M mutation in pediatric CNS tumors: a marker for diffuse high-grade astrocytomas. Am J Clin Pathol.

[9] Lewis PW, Muller MM, Koletsky MS, Cordero F, Lin S, Banaszynski LA, Garcia BA, Muir TW, Becher OJ, Allis CD (2013). Inhibition of PRC2 activity by a gain-of-function H3 mutation found in pediatric glioblastoma. Science.

[10] Chan KM, Fang D, Gan H, Hashizume R, Yu C, Schroeder M, Gupta N, Mueller S, James CD, Jenkins R, Sarkaria J, Zhang Z (2013). The histone H3.3K27M mutation in pediatric glioma reprograms H3K27 methylation and gene expression. Genes Dev.

[11] Louis DN, Perry A, Reifenberger G, von Deimling A, Figarella-Branger D, Cavenee WK, Ohgaki H, Wiestler OD, Kleihues P, Ellison DW (2016). The 2016 World Health Organization Classification of Tumors of the Central Nervous System: a summary. Acta Neuropathol.

[12] Panwalkar P, Clark J, Ramaswamy V, Hawes D, Yang F, Dunham C, Yip S, Hukin J, Sun Y, Schipper MJ, Chavez L, Margol A, Pekmezci M (2017). Immunohistochemical analysis of H3K27me3 demonstrates global reduction in group-A childhood posterior fossa ependymoma and is a powerful predictor of outcome. Acta Neuropathol.

[13] Bechet D, Gielen GG, Korshunov A, Pfister SM, Rousso C, Faury D, Fiset PO, Benlimane N, Lewis PW, Lu C, David Allis C, Kieran MW, Ligon KL (2014). Specific detection of methionine 27 mutation in histone 3 variants (H3K27M) in fixed tissue from high-grade astrocytomas. Acta Neuropathol.

[14] Venneti S, Santi M, Felicella MM, Yarilin D, Phillips JJ, Sullivan LM, Martinez D, Perry A, Lewis PW, Thompson CB, Judkins AR (2014). A sensitive and specific histopathologic prognostic marker for H3F3A K27M mutant pediatric glioblastomas. Acta Neuropathol.

[15] Pratt D, Natarajan SK, Banda A, Giannini C, Vats P, Koschmann C, Mody R, Chinnaiyan A, Venneti S (2018). Circumscribed/non-diffuse histology confers a better prognosis in H3K27M-mutant gliomas. Acta Neuropathol.

[16] Bender S, Tang Y, Lindroth AM, Hovestadt V, Jones DT, Kool M, Zapatka M, Northcott PA, Sturm D, Wang W, Radlwimmer B, Hojfeldt JW, Truffaux N (2013). Reduced H3K27me3 and DNA hypomethylation are major drivers of gene expression in K27M mutant pediatric high-grade gliomas. Cancer Cell.

[17] Castel D, Philippe C, Calmon R, Le Dret L, Truffaux N, Boddaert N, Pages M, Taylor KR, Saulnier P, Lacroix L, Mackay A, Jones C, Sainte-Rose C (2015). Histone H3F3A and HIST1H3B K27M mutations define two subgroups of diffuse intrinsic pontine gliomas with different prognosis and phenotypes. Acta Neuropathol.

[18] Wu G, Broniscer A, McEachron TA, Lu C, Paugh BS, Becksfort J, Qu C, Ding L, Huether R, Parker M, Zhang J, Gajjar A, Dyer MA (2012). Somatic histone H3 alterations in pediatric diffuse intrinsic pontine gliomas and non-brainstem glioblastomas. Nat Genet.

[19] Long W, Yi Y, Chen S, Cao Q, Zhao W, Liu Q (2017). Potential New Therapies for Pediatric Diffuse Intrinsic Pontine Glioma. Front Pharmacol.

[20] Hashizume R (2017). Epigenetic Targeted Therapy for Diffuse Intrinsic Pontine Glioma. Neurol Med Chir (Tokyo).

[21] Aboian MS, Solomon DA, Felton E, Mabray MC, Villanueva-Meyer JE, Mueller S, Cha S (2017). Imaging Characteristics of Pediatric Diffuse Midline Gliomas with Histone H3 K27M Mutation. AJNR Am J Neuroradiol.

[22] Venneti S, Garimella MT, Sullivan LM, Martinez D, Huse JT, Heguy A, Santi M, Thompson CB, Judkins AR (2013). Evaluation of histone 3 lysine 27 trimethylation (H3K27me3) and enhancer of Zest 2 (EZH2) in pediatric glial and glioneuronal tumors shows decreased H3K27me3 in H3F3A K27M mutant glioblastomas. Brain Pathol.

[23] Bugiani M, Veldhuijzen van Zanten SEM, Caretti V, Schellen P, Aronica E, Noske DP, Vandertop WP, Kaspers GJL, van Vuurden DG, Wesseling P, Hulleman E (2017). Deceptive morphologic and epigenetic heterogeneity in diffuse intrinsic pontine glioma. Oncotarget.

[24] Bayliss J, Mukherjee P, Lu C, Jain SU, Chung C, Martinez D, Sabari B, Margol AS, Panwalkar P, Parolia A, Pekmezci M, McEachin RC, Cieslik M (2016). Lowered H3K27me3 and DNA hypomethylation define poorly prognostic pediatric posterior fossa ependymomas. Sci Transl Med.

[25] Cleven AH, Sannaa GA, Briaire-de Bruijn I, Ingram DR, van de Rijn M, Rubin BP, de Vries MW, Watson KL, Torres KE, Wang WL, van Duinen SG, Hogendoorn PC, Lazar AJ (2016). Loss of H3K27 tri-methylation is a diagnostic marker for malignant peripheral nerve sheath tumors and an indicator for an inferior survival. Mod Pathol.

[26] Puget S, Beccaria K, Blauwblomme T, Roujeau T, James S, Grill J, Zerah M, Varlet P, Sainte-Rose C (2015). Biopsy in a series of 130 pediatric diffuse intrinsic Pontine gliomas. Childs Nerv Syst.

[27] Saratsis AM, Yadavilli S, Magge S, Rood BR, Perez J, Hill DA, Hwang E, Kilburn L, Packer RJ, Nazarian J (2012). Insights into pediatric diffuse intrinsic pontine glioma through proteomic analysis of cerebrospinal fluid. Neuro Oncol.

[28] Zheng PP, Luider TM, Pieters R, Avezaat CJ, van den Bent MJ, Sillevis Smitt PA, Kros JM (2003). Identification of tumor-related proteins by proteomic analysis of cerebrospinal fluid from patients with primary brain tumors. J Neuropathol Exp Neurol.

