# Correction: Detection of histone H3 K27M mutation and post-translational modifications in pediatric diffuse midline glioma via tissue immunohistochemistry informs diagnosis and clinical outcomes

**DOI:** 10.18632/oncotarget.26881

**Published:** 2019-04-12

**Authors:** Tina Huang, Roxanna Garcia, Jin Qi, Rishi Lulla, Craig Horbinski, Amir Behdad, Nitin Wadhwani, Ali Shilatifard, Charles James, Amanda M. Saratsis

**Affiliations:** ^1^ Division of Pediatric Neurosurgery, Department of Surgery, Ann & Robert H. Lurie Children’s Hospital of Chicago, Chicago, IL, USA; ^2^ Department of Neurological Surgery, Northwestern University Feinberg School of Medicine, Chicago, IL, USA; ^3^ Division of Pediatric Hematology/Oncology, Hasbro Children’s Hospital, The Warren Alpert Medical School of Brown University, Providence, RI, USA; ^4^ Department of Pathology, Northwestern University Feinberg School of Medicine, Chicago, IL, USA; ^5^ Department of Pathology, Ann & Robert H. Lurie Children’s Hospital of Chicago, Chicago, IL, USA; ^6^ Department of Biochemistry and Molecular Genetics, Northwestern University Feinberg School of Medicine, Chicago, IL, USA

**This article has been corrected:** The correct author information is given below:

**Amanda M. Saratsis**

Original article: Oncotarget. 2018; 9:37112-37124. https://doi.org/10.18632/oncotarget.26430

