# Supplementary material for: Detection of histone H3 K27M mutation and post-translational modifications in pediatric diffuse midline glioma via tissue immunohistochemistry informs diagnosis and clinical outcomes
Source: Oncotarget. 2018 Dec 14;9(98):37112–24. doi: 10.18632/oncotarget.26430 (PMC6324678; doi:10.18632/oncotarget.26430)
Supplement: Supplementary file 1 [file oncotarget-09-37112-s001.pdf]

Detection of histone H3 K27M mutation and post-translational modifications in pediatric diffuse midline glioma via tissue immunohistochemistry informs diagnosis and clinical outcomes

SUPPLEMENTARY MATERIALS

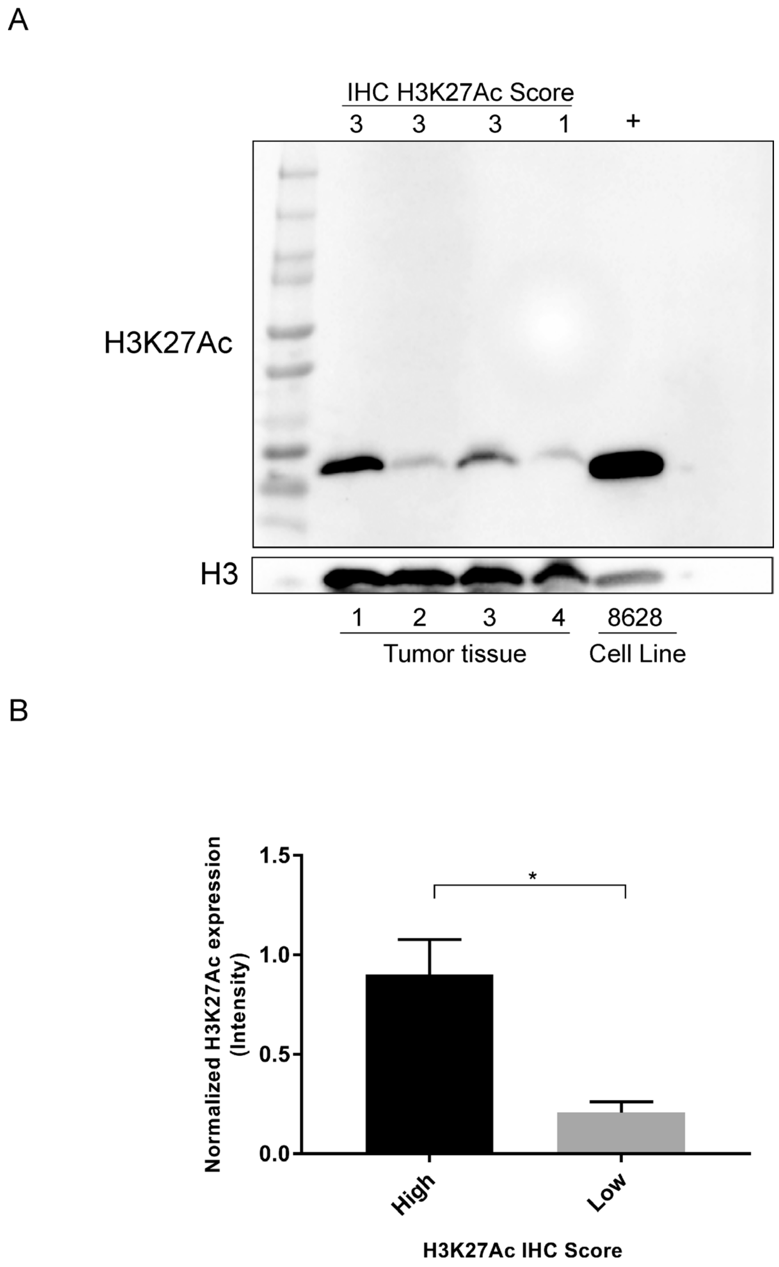

Supplementary Figure 1: Tissue H3K27Ac levels correlates with H3K27Ac IHC staining results. To validate tissue IHC results, western blot analysis of total protein extracted from fresh frozen tumor tissue specimens ($n = 4$) was performed in technical duplicate with the H3K27Ac antibody used for IHC staining. Protein from a well-characterized patient derived DIPG cell line, SF8628, was analyzed as a positive control, and total extracted Histone H3 protein used as a loading control (**A**). Densitometry analysis of resulting bands demonstrated higher relative H3K27Ac in tumor samples 1-3, which were given a H3K27Ac IHC score of 3, relative to sample 4, which was assigned a H3K27Ac IHC score of 1 ($p = 0.0357$, Mann–Whitney U test), (**B**). Band intensity was normalized to total H3, error bar represents standard deviation.
